# Client preferences and acceptability for medical abortion and MVA as early pregnancy termination method in Northwest Ethiopia

**DOI:** 10.1186/1742-4755-8-19

**Published:** 2011-06-03

**Authors:** Mulatu A Woldetsadik, Tegbar Y Sendekie, Mary T White, Desalegn T Zegeye

**Affiliations:** 1 Department of Gynaecology and Obstetrics, College of Medicine and Health Sciences, University of Gondar, Gondar, P.O.Box-196, Ethiopia; 2 JHPIEGO Ethiopia, Addis Ababa, Ethiopia; 3 Department of Community Health, Wright State University, Dayton, USA; 4 Department of Epidemiology, College of Medicine and Health Sciences, University of Gondar, Gondar, P.O.Box-196, Ethiopia

## Abstract

**Background:**

Increasing access to safe abortion services is the most effective way of preventing the burden of unsafe abortion, which is achieved by increasing safe choices for pregnancy termination. Medical abortion for termination of early abortion is said to safe, effective, and acceptable to women in several countries. In Ethiopia, however, medical methods have, until recently, never been used. For this reason it is important to assess women's preferences and the acceptability of medical abortion and manual vacuum aspiration (MVA) in the early first trimester pregnancy termination and factors affecting acceptability of medical and MVA abortion services.

**Methods:**

A prospective study was conducted in two hospitals and two clinics from March 2009 to November 2009. The study population consisted of 414 subjects over the age of 18 with intrauterine pregnancies of up to 63 days' estimated gestation. Of these 251 subjects received mifepristone and misoprostol and 159 subjects received MVA. Questionnaires regarding expectations and experiences were administered before the abortion and at the 2-week follow-up visit.

**Results:**

The study groups were similar with respect to age, marital status, educational status, religion and ethnicity. Their mean age was about 23, majority in both group completed secondary education and about half were married. Place of residence and duration of pregnancy were associated with method choice. Subjects undergoing medical abortions reported significantly greater satisfaction than those undergoing surgical abortions (91.2% vs 82.4%; *P *< .001). Of those women who had medical abortion, (83.3%) would choose the method again if needed, and (77.4%) of those who had MVA would also choose the method again. Ninety four percent of women who had medical abortion and 86.8% of those who had MVA would recommend the method to their friends.

**Conclusions:**

Women receiving medical abortion were more satisfied with their method and more likely to choose the same method again than were subjects undergoing surgical abortion. We conclude that medical abortion can be used widely as an alternative method for early pregnancy termination.

## Background

Unsafe abortion is a public health problem. Globally, 20 million unsafe abortions take place each year and account for 13% of all maternal deaths [1]. In Ethiopia 32% of all maternal death is due to unsafe abortion [2]. In addition, unsafe abortion accounts for nearly 60% of all gynecologic admissions and almost 30% of all obstetric and gynecologic admissions in Ethiopia [2,3]. In Ethiopia, as of May 2005, the law on abortion was revised in Article 551 of the Penal Code, to include four legal grounds in which pregnancy can be terminated: rape or incest, lethal congenital malformation, physical and mental deficiency to bring up the child, and if continuation of pregnancy endangers the life of the mother or the child or the health of the mother [4]. According to the law, no consent from a spouse, partner or parent is required to obtain a legal abortion and no requirements exist for legal reporting or documenting rape or incest as a prerequisite for obtaining a legal abortion [4].

Based on the revised law, the Federal Ministry of Health of Ethiopia developed technical and procedural guidelines for safe abortion services [2]. According to the national guidelines on safe abortion services, the Family Health Department of the Ministry of Health of Ethiopia, and partners like Ipas are collaborating to expand access to comprehensive abortion services. The introduction of low cost pre-packaged preparation of mifepristone and misoprostol, Medabon^® ^is underway. The preparation consists of 200 mg mifepristone (one tablet), to be taken on day 1, and 0.8 mg misoprostol (four tablets) to be taken on day 2 or day 3.

When properly administered, abortion is one of the safest procedures in contemporary medical practice. So, increasing access to safe abortion services is the most effective way of preventing the burden of unsafe abortion [5,6].

For a new technology to be a viable option in a given health care system, even when services are free, people need to want to use it and to demand it. For this reason, understanding how potential patients perceive and value a new technology is important [7,8]. In the case of medical abortion, however, patient acceptability is key to the success of the method [6,7,9-11]. If a procedure is acceptable, woman would choose it again if they needed to terminate another pregnancy and would recommend it to their friends [12]. Indeed, the combination of mifepristone-misoprostol for early abortion is established as safe, effective, and acceptable to women in several developing countries. In Ethiopia, however there was no study conducted on the choice and preference of medical method of abortion and MVA.

For this reason, it is timely and important to assess women's preferences for, and the acceptability of, medical abortion and MVA in the early first trimester pregnancy termination and factors respectively affecting the acceptability of medical and MVA abortion services.

## Methods

A prospective study was conducted from March 2009 to November 2009. The study was conducted in 4 health institutions located in North Gondar, East and West Gojjam, where both medical abortion and MVA were provided. Four health institutions, namely, Gondar University Hospital, DebreMarkos Hospital, Marystopes clinics of DebreMarkos and Bahirdar were included. Other Government Hospitals and health institutions were not selected because the service was not fully started during the data collection period. Sample size of 414 was reached for the study, assuming greater than 10,000 eligible women will seek to terminate pregnancy in the selected institutions during the study period, 5% of level of significance, 5% margin of error, and that 57.2% of clients prefer medical method of terminating pregnancy [8]. Expecting a non-response rate of 10% (since abortion is sensitive issue) and maintaining high statistical power, the final required sample size became 414 women. Women with gestational age ≤63 days from their last menstrual period, with abdominal ultrasound or by physical examination ≤9 weeks, who sought abortion and fulfilled the criteria of the Revised Abortion Law of Ethiopia [4], and who came during the study period, were eligible for the study. Those who had contraindications to medical or surgical abortion were excluded from the study. Those who were not able to provide information due to language barriers or cognitive incapacitation were also excluded.

All clients who requested termination of early pregnancy were given the same oral information about the two methods without a recommendation of one method over the other.

The medical abortion protocol consisted of 200 mg mifepristone at the first visit, followed by 800-μg vaginal misoprostol 48 hours later, and a follow up visit scheduled after 14 days. Backup surgical abortion was provided to women experiencing medical method failure and complications. Women in the surgical group of the study received the standard procedure of vacuum aspiration with local anaesthesia. Follow up visit for a surgical client was scheduled 14 days after the procedure. All procedures were done by trained providers.

Eight data collectors who were nurses in the respective facilities but not on duty received training for one day collect the data. A pretested and structured interview questionnaire was used as the instrument of data collection. Data collected at the first visit were on demographic characteristics and reasons for choice of the procedure. A follow up questionnaire assessed the actual abortion experience. Data were collected on overall satisfaction, future choice of an abortion method, acceptability (acceptability of medical and MVA abortion in this study is re-use/recommendation to a friend), side effects, and place of possible pregnancy expulsion.

Quality of data was assured by using a structured questionnaire, which was translated to Amharic and pre-tested before the actual survey, and by training data collectors prior to the survey. In addition, regular supervision and checking filled data for completeness, clarity and accuracy was made every day by the investigators.

Data were coded, entered and cleaned, using SPSS version 13 statistical package. Some variables were recorded into different variables then analysis was done. Frequencies, percentages, cross tabulations and Chi-square tests were used for socio demographic and obstetrics characteristics, method choice and reasons for selecting the method, acceptability, abortion experience and satisfaction. In order to test for the associations of the outcome variables against the independent variable multiple logistic regressions was applied accordingly the p-value, confidence interval and odds ratio were computed and interpreted.

Ethical clearance for the study was obtained from the Institutional Review Board of the University of Gondar. The study subjects were informed about the purpose of the research, the benefit and the risk of participation, the privacy and confidentiality of the study and their right to withdraw at any time from the study and this had no effect on their treatment. Verbal consent was obtained from each subject.

## Results

A total of 414 women were included in the study. Four incomplete questionnaires were excluded from analysis.

The study groups were similar with respect to age, marital status, educational status, religion and ethnicity. The mean age of women who opted for the medical method was 23.1 years (range: 15-40 years) and that of MVA was 23.7 years (range: 15-43 years) respectively. However, more women from urban areas (64%) chose medical abortion as compared to the rural residents (51%). (Table 1).

**Table 1:** Socio-demographic characteristics of the respondents by method choice in four selected health institutions, Northwest Ethiopia, November, 2009

Variable	Medical	MVA
**Age (mean)**	23 ± 4.4	23.7 ± 4.8
Place of residence*		
Urban	204(81.3)	115(72.3)
Rural	47(18.7)	44(27.7)
**Marital status**		
Single	122(48.6)	71(44.7)
Married	119(47.4)	84(52.8)
Other	10(4.0)	4(2.5)
**Educational level**		
Illiterate	37(14.7)	35(22.0)
Primary	40(15.9)	31(19.5)
Secondary and above	174(69.3)	93(58.5)
**Religion**		
Orthodox	236(94.0)	144(90.6)
Muslim	10(4.0)	12(7.5)
Other	5(2.0)	3(1.9)
**Ethnicity**		
Amhara	245(97.6)	153(96.2)
Oromo	4(1.6)	3(1.9)
Other	2(.8)	3(1.9)

*P < 0.02

The majority of study participants had no child previously (67.3% of women who chose medical abortion and 59.7% of women who chose MVA). About 85% of both groups reported no history of previous abortion. The duration of pregnancy was less than 7 weeks in 174(69.3%) women who chose medical abortion and 89(56.0%) of those who chose MVA (Table 2)

**Table 2:** Obstetrics characteristics of the respondents by method choice in four selected health institutions, Northwest Ethiopia, November, 2009

Variable	Medical	MVA
Nulliparae	67.3%	59.7%
Number of children (mean ± sd)	2 ± 1.3	2.2 ± 1.7
Ever had abortion	14.7%	15.1%
Duration of current pregnancy* (mean ± sd)	46 ± 8.9	49 ± 8.9

*p < 0.02

### Method choice

Overall 251(61.2%) women chose medical abortion and 159(38.8%) opted for MVA. Among women who selected the medical method, 120(47.8%) did so primarily to avoid pain and 19(7.6%) chose to avoid surgery or anesthesia. In contrast, women choosing surgical abortion did so mainly because it entailed fewer visits 102(64.2%) and was believed to be safer 80(31.9%) than the medical method of pregnancy termination (Table 3).

**Table 3:** Choice of method and the reasons for selecting their method of respondents in four selected health institutions, Northwest Ethiopia, November, 2009

Variable	Percentage
**Reason to choose medical**	
To avoid pain	47.8
Safer	31.9
To avoid surgery or anesthesia	7.6
Fear of side effect	6.8
Others	6
**Reason to choose MVA**	
Fewer visit	64.2
Simpler and faster	20.8
To avoid side effect	6.3
More effective	7.5
Others	1.3

Binary logistic regression was conducted to examine factors associated with method choice. Those women who were living in urban area were 1.64 times more likely to choose medical abortion than MVA and women with duration of pregnancy less than 49 days were 1.77 times more likely to choose medical abortion than MVA (not shown on table).

### Acceptability and abortion experience

There were 13(5.2%) failures of medical abortion and 7(4.4%) MVA failures. Of the side effects, abdominal cramp, nausea and vomiting were common in both methods but more common in medical than MVA. With regard to place of abortion 182(76.5%) of medical abortions took place at home compared with 3(2.0%) of MVA. The majority of women had less than seven days of bleeding in both methods of abortion (Table 4).

**Table 4:** Abortion experience of respondents by method in four selected health institutions, Northwest Ethiopia, November 2009

Variable	Medical	*MVA*
**Pregnancy Expelled**	94.4%	92.5%
**Place of abortion**		
Health facility	50(21%)	144(98%)
Home	182(76.5%)	3(2%)
Could not identify location	4(2.1%)	
Other	1(0.4%)	
Duration of bleeding*(mean ± sd)	6.7**± 5.2**	3.6**± 3.7**
**Side effects**		
Abdominal cramp*	85.7%	66%
Nausea*	56.2%	27.7%
Vomiting*	25.1%	15.1%
Diarrhea	3.6%	0.6%
Fever	11.6%	6.3%
Headache	13.1%	15.7%

*P < 0.01

The majority of women were satisfied with their abortion experience, but those women who had medical abortion were more satisfied than MVA (91.2% vs. 82%). Most women who received abortion services said they would choose the method again should the need arise, 83.3% medical abortion and 77.4% MVA respectively. Nearly all (94%) medical abortion clients and 86.8% of MVA abortion clients would recommend their method to their friends. However, there was no significant difference between medical abortion and MVA users with regard to future choice of the method and recommendation of the method to friends (Table 5).

**Table 5:** Distribution of abortion patients, by measure of satisfaction and acceptability with their method, according to method in four selected health institutions, Northwest Ethiopia, November, 2009

Variable	Medical	MVA
**Satisfaction**		
Satisfied with the method*	91.2%	82.4%
**Reason for dissatisfaction**		
Heavy bleeding	7(31.8%)	5(17.9%)
Method failure	10(45.5%)	7(25%)
Pain	5(22.7%)	16(57.1%)
**Will choose the method again**	83.3%	77.4%
Will recommend the method *	94%	86%

*p < 0.05

## Discussion

This study of 410 women represents the first report for early first trimester pregnancy termination undertaken in Ethiopia. In our study, 251(61.2%) women chose medical method. This is consistent with another study in Vietnam where 66.2% of subjects preferred medical abortion and 33.8% opted for MVA [13]. Similarly, in the United Kingdom, 57% preferred medical and 42.8% opted for MVA [8]. Most women living in urban areas and those women in early pregnancy opted for medical abortion over MVA in our study. The reason could be those women who were living in rural areas want to avoid repeated visit to the health institution. This finding supported by similar study done in India where medical abortion was more commonly opted by women belonging to urban areas and in early pregnancy (86.6% vs 46.6%) [14].

Regarding the location of the abortion, 76.5% of medical abortion took place at home. The reason could be women were not kept at health institution for the first 4 to 6 hours after taking mifepristone by which 90% of abortion occurs. In addition to this, in our study the total duration from administration of misoprostol to possible expulsion of pregnancy was not collected. This finding is different from similar study done in Vietnam were 72% of medical abortion took place in the clinic [13].

In our study, 91.2% of women using the medical method and 82.4% of women using MVA were satisfied. Similarly, 83.3% of those who used the medical method and 77.4% of those who used MVA would opt for the same method of treatment if they were ever to have another termination of pregnancy. However, there was no statistically significant difference among the two groups. The reason why the medical method was not selected by all in our study could be side effects like abdominal pain, nausea and vomiting were more common in medical than MVA client. A study done in Vietnam showed women who had medical abortion were more likely to select the same method again than were those who selected MVA(96%vs.52%) [13].

With regard to recommendation of the method to their friends, those women who chose medical abortion would recommend their method slightly more often compared with those who choose MVA (94% vs86.8%). This may be due to those women who had abdominal pain were less likely to recommend MVA to their friends, but this had no influence on recommendation of medical method to friends. Our results are similar to studies done in Finland, Denmark and UK [8,11,15].

In our study side effects like abdominal pain and nausea affected acceptability of both methods, hence counselling about women's expectations and appropriate pain control should be provided.

In this study some of the findings had wide confidence interval; this could be due to small number of cases in some of the cells.

## Conclusions

This study showed that medical method for termination of early pregnancy was chosen more compared with MVA and the choice of medical abortion was associated with duration of pregnancy and place of residence. Similarly, both medical abortion and MVA were accepted and most women were satisfied by both methods. Acceptability may be affected by side effects of abortion methods.

## Competing interests

The authors declare that they have no competing interests.

## Authors' contributions

MAW wrote the proposal, participated in data collection, analyzed the data and drafted the paper. DTZ, TYS and MTW approved the proposal with some revisions, participated in data analysis and revised subsequent drafts of the paper. All authors read and approved the final manuscript.
